# Recognition of fungal priority pathogens: What next?

**DOI:** 10.1371/journal.pntd.0011136

**Published:** 2023-03-09

**Authors:** Marcio L. Rodrigues, Joshua D. Nosanchuk

**Affiliations:** 1 Instituto Carlos Chagas, Fundação Oswaldo Cruz (Fiocruz), Curitiba, Brazil; 2 Instituto de Microbiologia Paulo de Góes (IMPG), Universidade Federal do Rio de Janeiro, Rio de Janeiro, Brazil; 3 Department of Medicine (Division of Infectious Diseases) and Department of Microbiology and Immunology, Albert Einstein College of Medicine, New York, New York, United States of America; Duke University School of Medicine, UNITED STATES

Fungal diseases kill more than 1.5 million people per year, mostly in regions where neglected populations live [1]. This alarming number of deaths is part of a highly complex problem, since mortality alone does not give a complete picture of the fungal burden in different populations [2]. Indeed, several fungal diseases are associated with low mortality but directly linked to social exclusion and hospitalization. For instance, patients suffering from chromoblastomycosis manifest chronic and progressive cutaneous and subcutaneous lesions that can persist for decades [3]. Affected individuals may have significant mental health issues associated with this rare and chronic condition [4]. Fungal infections are the most diagnosed skin disease in Africa, especially in 1- to 5-years-old children [5], and these cutaneous mycoses are associated with social disapproval and psychological trauma, negatively affecting their performance in class [6]. These and other examples demonstrate that the burden of fungal infections is not restricted to mortality. Another way of measuring the impact of fungi on public health is to determine their overall burden of disease using the disability-adjusted life year (DALY). One DALY represents the loss of the equivalent of 1 year of full health [2]. However, with a few exceptions [5,7–10], DALY estimates are not available for most of the fungal diseases.

Although fungal diseases are directly associated with neglected populations, they clearly also cause important public health problems in developed countries. For example, in the United States, individuals with diseases such as coccidioidomycosis experience 3 or more months of diminished mental and emotional health [11]. The economic impact of fungal diseases is remarkable. For example, in 2019, the US spent approximately USD 11.5 billion on serious fungal diseases, distributed into direct medical costs, productivity loss due to absenteeism, and premature deaths [12]. To give this number tangible dimensions, USD 11.5 billion represent 25% of the annual budget of the US National Institutes of Health (NIH) [13]. Even diseases such as onychomycosis cost over USD 2,000 per person affected in antifungal drug expenses alone [14]. In large part due to the complexity of fungi, including that they are eukaryotic organisms, the pace of generation of knowledge in the area of fighting fungal diseases is clearly slower than other areas [15]. There are no human licensed antifungal vaccines, and much has been discussed about the problems of antifungal therapies, which include high costs, emergence of antifungal resistance, significant toxicity, and limited number of antifungal drug classes [16]. It is likely that this complex scenario is the consequence of reduced funding for research and insufficient recognition of the impact of fungal diseases on public health by science policymakers and health authorities [17]. This scenario contrasts with the ever-changing epidemiology of fungal infections, which includes the emergence of highly lethal and drug-resistant pathogenic species [18]. Finally, the Coronavirus Disease 2019 (COVID-19) pandemic exacerbated the epidemiology of fungal diseases [19], proving the concept that globally transmitted diseases can make worse an already complex epidemiologic scenario.

Several initiatives to reduce deaths caused by fungal diseases have been proposed in recent years [20,21]. Still, the impact of fungal diseases on public health and economy remains unacceptably high. As an attempt to attract attention to the importance of fighting fungal infections, the World Health Organization (WHO) launched a list of priority fungal pathogens in October 2022 [22]. This list, defined by WHO as “the first global effort to systematically prioritize fungal pathogens, considering their unmet research and development needs and perceived public health importance,” was divided into 3 categories: critical, high, and medium priority. Three primary areas for action were proposed, focusing on (1) strengthening laboratory capacity and surveillance; (2) sustainable investments in research, development, and innovation; and (3) public health interventions. This is, unquestionably, a commendable action. We will add to the WHO report our view on additional initiatives that may contribute to strengthening the medical mycology community and, consequently, the qualified generation of knowledge in this area, which will likely culminate in the development of innovative tools to prevent, control, and diagnose fungal diseases.

## 1. Generation of knowledge in medical Mycology

There is an ongoing debate on the allocation of science funding. While much of the scientific community in universities and scientific foundations is focused on fundamental sciences, there is an undeniable call from the general public for making science more translational. The SARS-CoV-2 pandemic clearly demonstrated that orchestrated initiatives to foster innovation can produce concrete tools mostly for diagnosing and preventing COVID-19. However, it is important to highlight that the most positively disruptive innovation in the pandemic—RNA-based vaccines—originated from a combination of fundamental discoveries [23]. In a broader analysis, a relatively recent study on the 28 most transformative medicines approved for clinical use by the US Food and Drug Administration (FDA) between 1985 and 2009 revealed that approximately 80% of the medicines in this group could be traced back to one or several basic discoveries [24]. Therefore, although innovation-oriented programs are of unquestionable relevance, continued fostering of fundamental research is indispensable to generate translational tools.

The 19 fungal pathogens included in the WHO priority list are listed in Table 1. This classification is based on the WHO estimates of the weight of each pathogen according to perceived public health importance [22]. Based on this estimate, other medically important fungi are not included in the priority list, although their impact on public health is not questionable. The genera *Sporothrix*, *Blastomyces*, *Fonsecaea*, *Emergomyces*, and *Cladosporium* are examples of excluded fungi that are medically important but still neglected fungal pathogens, as highlighted by the ongoing epidemic of sporotrichosis in Brazil [25,26]. Similarly, the incidence of blastomycosis has increased in recent years. As illustrated by the Minnesota Department of Health, 82 cases were reported in the state in 2021. Most years, 9% of people with blastomycosis die from the infection, but in 2021, 23% of patients died [27]. This picture becomes even more complex if extremely rare fungal infections are considered. For instance, central nervous system infections caused by brown-black fungi still have high mortality rates, regardless of underlying immune status or treatment approaches [28].

**Table 1:** WHO fungal pathogens priority list.

Fungal pathogen	Priority classification
*Aspergillus fumigatus*	Critical
*Candida albicans*	
*Candida auris*	
*Cryptococcus neoformans*	
*Candida parapsilosis*	High priority
*Candida tropicalis*	
Eumycetoma causative agents	
*Fusarium* spp.	
*Histoplasma* spp.	
Mucorales	
*Nakaseomyces glabrata* (*Candida glabrata*)	
*Coccidioides* spp.	Medium priority
*Cryptococcus gattii*	
*Lomentospora prolificans*	
*Paracoccidioides* spp.	
*Pichia kudriavzeveii* (*Candida krusei*)	
*Pneumocystis jirovecii*	
*Scedosporium* spp	
*Talaromyces marneffei*	

**Fig 1:**
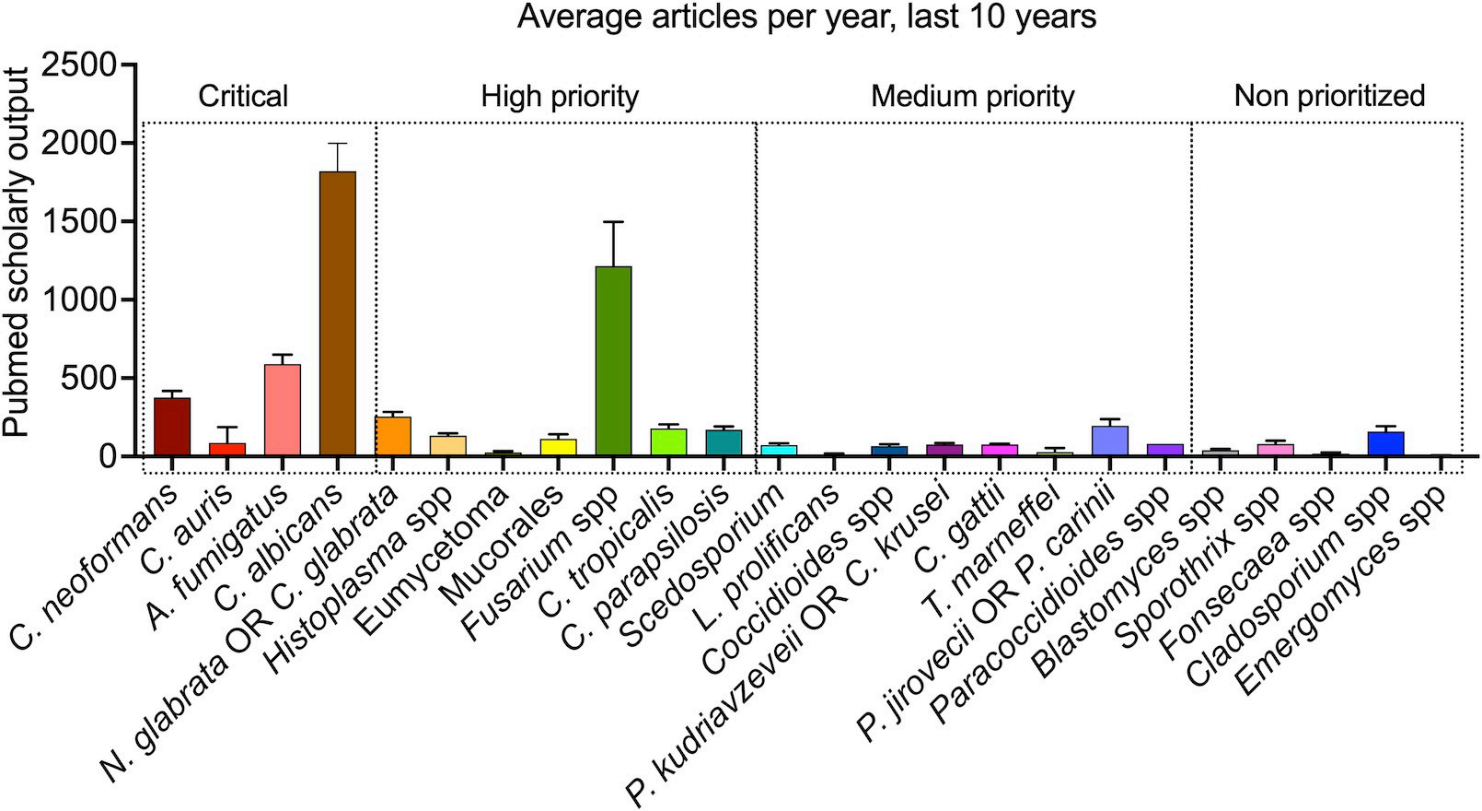
Scholarly output (2012 to 2021, Pubmed documents) involving the fungal pathogens prioritized by WHO or those not included in the WHO priority list (classifications are listed over the boxed areas). Search terms corresponded to those listed in the *x*-axis.

**Fig 2:**
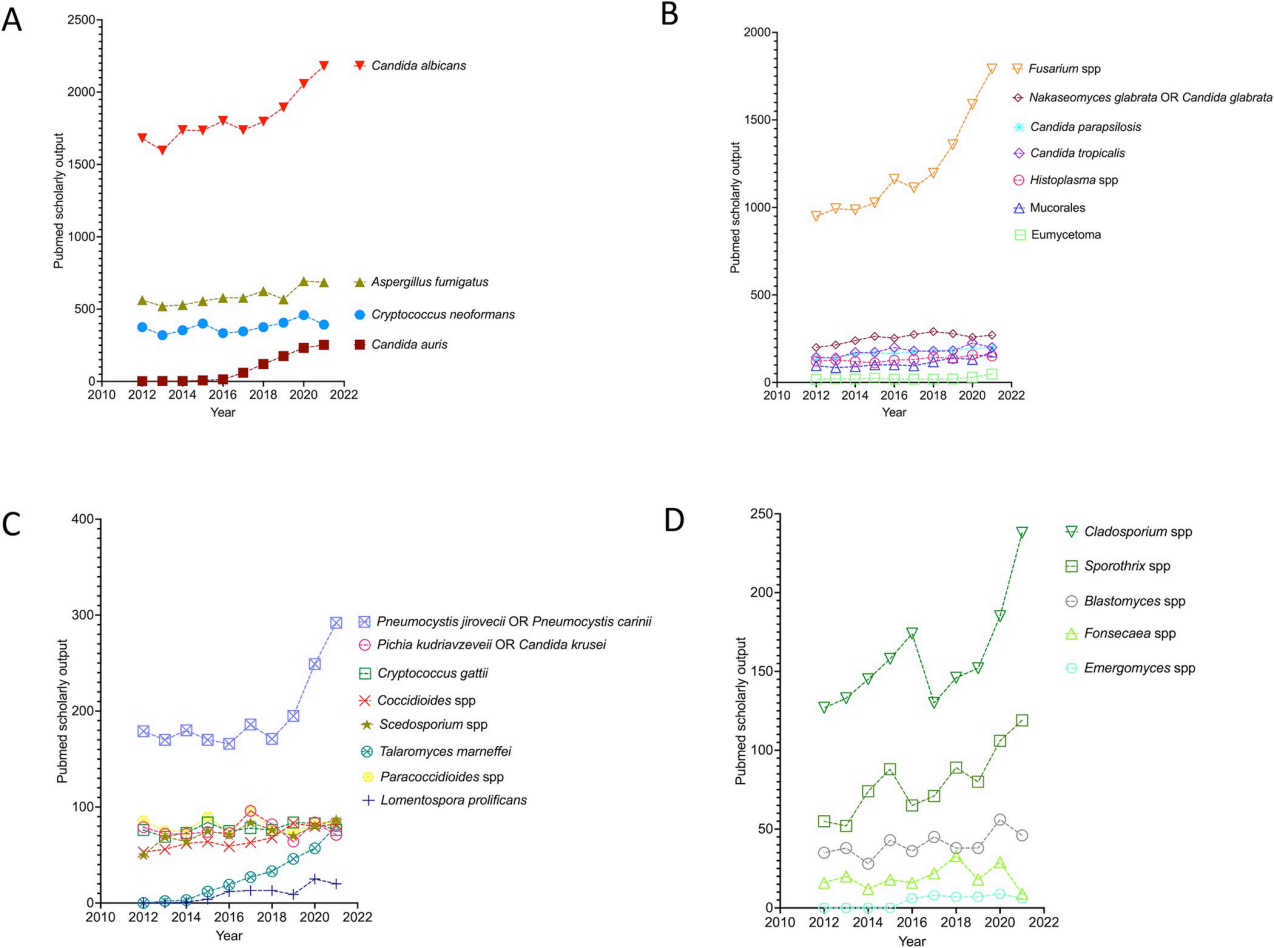
Yearly scholarly output (2012 to 2021, PubMed documents) involving the fungal pathogens listed by WHO as critical (**A**), high (**B**), or medium (**C**) priority, in addition to those belonging to genera that were not prioritized by WHO (**D**). Search terms corresponded to those listed on the right side of each panel.

As an illustration of the pace of knowledge generation in the fight of fungal infections, the publication rates in the area of antifungal research are considerably lower than those found, for instance, in antibacterial research [15]. We performed simple PubMed searches (2012 to 2021) using the terms listed by WHO or the additional pathogens listed above (fungal species or genus) (Fig 1). This analysis suggests that the generation of knowledge (translated by scholarly output) in the area of fungal diseases is highly variable and apparently not correlated with mortality or DALYs, since publication patterns suggesting an association with the WHO list or public health impact were not identifiable. A more detailed analysis of the critical group during the last decade demonstrated that the scholarly output has constantly increased in the 2 *Candida* species, while it tended to be flat in *A*. *fumigatus* and *C*. *neoformans* (Fig 2A). In the high-priority group, the number of publications per year remained stable over time in all pathogens except *Fusarium* (Fig 2B). However, the *Fusarium* numbers were inflated by the ability of members of this genus to infect plants, resulting in higher (and health-unrelated) scholarly output. In the medium-priority group, all fungi trended to stability, with the emerging pathogens *C*. *gattii* and *P*. *jirovecii* representing the only exceptions (Fig 2C). Within the nonprioritized pathogens, scholarly output tended to grow over the years for *Cladosporium* spp. and *Sporothrix* spp., with no signs of expansion for *Blastomyces*, *Fonsecaea*, and *Emergomyces* (Fig 2D). In summary, the generation of knowledge has remained stable over the last decade for approximately 70% of pathogens approached here, independently on their position in the WHO list.

## 2. Innovation to prevent, control, and diagnose fungal diseases

Funding for fungal research is smaller than other areas with similar impact on public health [16,17,29], and innovation-oriented initiatives to fight fungal diseases are in fact uncommon. Recognizing the importance of neglected diseases is extremely important to generate innovative tools, and successful cases are already available. For instance, WHO established a goal of interrupting human African trypanosomiasis caused by *Trypanosoma brucei gambiense* by 2030 [30], which led to initiatives of drug development. In this context, acoziborole was selected in 2009 by the Drugs for Neglected Diseases initiative (DNDi) as a preclinical candidate to treat sleeping sickness caused by *T*. *b*. *gambiense* [31]. In 2012, acoziborole entered clinical development, and recently, the drug showed high efficacy and favorable safety profile, supporting its use in the fight against human African trypanosomiasis [32]. This is an efficient illustration of how effective the establishment of thematic initiatives to fight neglected diseases can be.

After the introduction of echinocandins into the clinic [33], innovative tools to fight mycoses remained restricted to a few diagnostic tests for almost 20 years [34]. However, important progress has been made in recent years [35,36]. The triterpenoid ibrexafungerp, an inhibitor of 1,3-beta-D-glucan synthase, was approved by the US FDA in June 2021 for the treatment of vaginal infections with *Candida* [37]. Olorofim, which received a breakthrough therapy designation from the FDA [38], is in the process of FDA approval as a potential anti-*Aspergillus* candidate since it shows activity against azole-resistant strains [35] as well as other difficult to treat fungi. Cochleated amphotericin is completing a Phase II trial for cryptococcal meningitis [35]. Similarly successful experiences have been reported in the field of diagnosis. For instance, diagnostics for cryptococcal meningitis in association with HIV have evolved in 10 years from culture and microscopic observation to detection of cryptococcal antigen (CrAg) [39]. CrAg titer predicts meningitis and death, and the recent development of a point-of-care lateral flow assay and its distribution into resource-limited laboratory settings has changed the way cryptococcosis is diagnosed and treated [39]. More recently, a rapid test to diagnose all clinical forms of the sporotrichosis was developed based on the detection of antibodies in the patients’ blood serum [40], which might impact the clinical decisions on how this disease will be treated. Although these are achievements of unquestionable importance, several fungal diseases remain in the need of novel therapeutic and/or diagnostic tools, denoting the need for innovation in the area of fungal infections.

### 3. Changing medical Mycology

Fundamental science is one of the motors of innovation, as already discussed in this manuscript. However, there are no reasons to believe that the generation of knowledge in the area of pathogenic fungi will be improved without programs to stimulate research in the field of fungal diseases. We suggest that funding agencies and leading journals can explore thematic calls to specifically address the fight against fungal diseases, such as done by the US NIH with the development of coccidioidomycosis collaborative research centers through RFA-AI-20-056 in 2020 to 2021. However, it is essential to expand these calls beyond the WHO priority list in order to avoid the risk of keeping other fungi of medical importance underappreciated and underexplored.

As discussed for other neglected diseases, a combination of academic, governmental, and private initiatives could be game-changing for fighting and/or preventing mycoses. For example, Caskey proposed ways to facilitate drug development to treat neglected diseases [41]. Increased investments in technology focused on improving target validation and drug safety should be made available to the academic research community, in addition to government research funding aimed at addressing health challenges. Simplification of regulatory steps are similarly relevant to facilitate the decisions of regulatory agencies and investors. Combined efforts between academia and the pharmaceutical industry are necessary too, possibly with the participation of funding agencies and even WHO in the decision process of go/no-go steps required for drug development. Programs including diagnostic tools and vaccine development that were successful for other areas of healthcare could also be replicated for fungal diseases. For instance, the global effort to develop and distribute effective vaccines against the COVID-19 coronavirus disease has produced several safe and effective options, and COVAX was established as the pillar of vaccine development, production, and equitable access of the Access to COVID-19 Tools Accelerator [42]. In the fight against Ebola, investments made by the US National Institute of Allergy and Infectious Diseases (NIAID), through Biodefense and Emerging Infectious Diseases Program funding, and by the Department of Defense (DoD) Defense Threat Reduction Agency (DTRA), through the Chemical and Biological Defense Program (CBDP), have led to the development of a majority of the countermeasures (drugs, vaccines, and diagnostics) in the pipeline [43]. Global efforts to fight fungal diseases could follow similar approaches.

### 4. Perspectives

The awareness of the importance of fungal diseases has unquestionably increased in the last decade. The recognition by WHO that several mycoses are neglected [44] and the launch of the pathogens’ priority list [22] represent major advances as do other initiatives such as the US Centre for Disease Control’s fungal diseases awareness week (https://www.cdc.gov/fungal/awareness-week.html), the Global Action For Fungal Infections (GAFFI; https://gaffi.org/) and others. GAFFI’s advocacy work and the CDC’s fungal awareness week are especially important to highlight is that education of the public, healthcare providers, and scientists about the fungal scourges adversely affecting our global community is essential to combat the diverse. The WHO paper also calls for the facilitation of international coordination, and this includes bringing together policymakers, healthcare providers, and scientists of richly diverse backgrounds to improve our response to these complex threats to human health. On the other hand, the possibility that the WHO list can produce negative effects needs attention. For instance, fungal pathogens that are considered medium priority or omitted from the list might become even more neglected and more difficult to receive funding. Given that over 6 billion people are estimated to have fungal diseases with up to 30% considered serious fungal infections [45], now is the time for concrete actions to fight a neglected problem that deeply impacts public health.
